# Foster Kennedy Syndrome Due to Meningioma Growth during Pregnancy

**DOI:** 10.3389/fneur.2013.00183

**Published:** 2013-11-11

**Authors:** Federico Rodríguez-Porcel, Ian Hughes, Douglas Anderson, John Lee, José Biller

**Affiliations:** ^1^Department of Neurology, Stritch School of Medicine, Loyola University Chicago, Maywood, IL, USA; ^2^Department of Pathology, Stritch School of Medicine, Loyola University Chicago, Maywood, IL, USA; ^3^Department of Neurosurgery, Stritch School of Medicine, Loyola University Chicago, Maywood, IL, USA

**Keywords:** Foster Kennedy syndrome, meningioma, pregnancy, optic disk atrophy, papilledema, anosmia

## Abstract

Tumors of the olfactory groove may cause unilateral optic atrophy with contralateral papilledema and anosmia (Foster Kennedy syndrome). We describe a case of a young pregnant woman with Foster Kennedy syndrome due to an olfactory groove meningioma.

## Case Presentation

A 37-year-old right handed woman from Rwanda in her 37th pregnancy week presented to our Emergency Department in labor. She was admitted to the High Risk Maternal Fetal Medicine Service. Neurology was consulted for assessment of protracted headaches of approximately 2 years duration, worsening since the first trimester of her pregnancy. In addition, visual acuity of her left eye (OS) had gradually worsened during the same period of time, resulting in complete monocular visual loss.

She had no history of nausea, vomiting, transient visual obscurations, galactorrhea, rhinorrhea, or seizures. She had no exposure to ionizing radiation or repeated cranial trauma.

**Figure 1:**
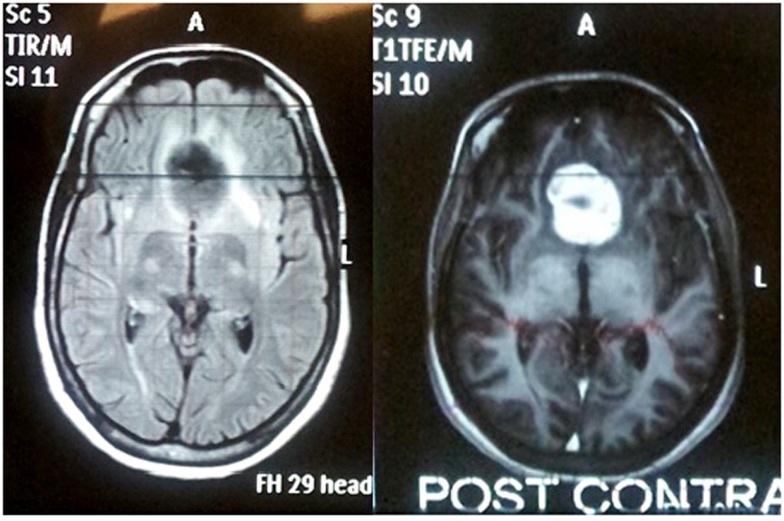
**MRI of the brain with and without contrast done in Rwanda 1 year previous to admission to our institution showed 3 cm × 2.6 cm × 1.2 cm homogeneously enhancing suprasellar mass along the dural floor of the cranial fossa**.

On neurologic examination she had bilateral anosmia, lack of light perception OS, and visual acuity was 20/20 OD. There was a left relative afferent pupillary defect (APD). Funduscopy showed pallor of the left optic disk and blurred nasal margins of the right optic disk. Complete blood count (CBC), complete metabolic profile (CMP), thyroid stimulating hormone (TSH), prolactin, follicular stimulating hormone, luteinizing hormone (LH), and growth hormone (GH) were within the normal range expected during pregnancy. MRI of the brain without contrast showed a 3.7 cm × 3.2 cm × 1.4 cm homogeneously isointense on T1 suprasellar mass along the dural floor of the cranial fossa with compression of the left optic apex with moderate edema and no hydrocephalus. This was a significant increase from her previous MRI done a year earlier in Rwanda (Figure 1). After the first MRI she was told to come to the US to seek neurosurgical intervention.

**Figure 2:**
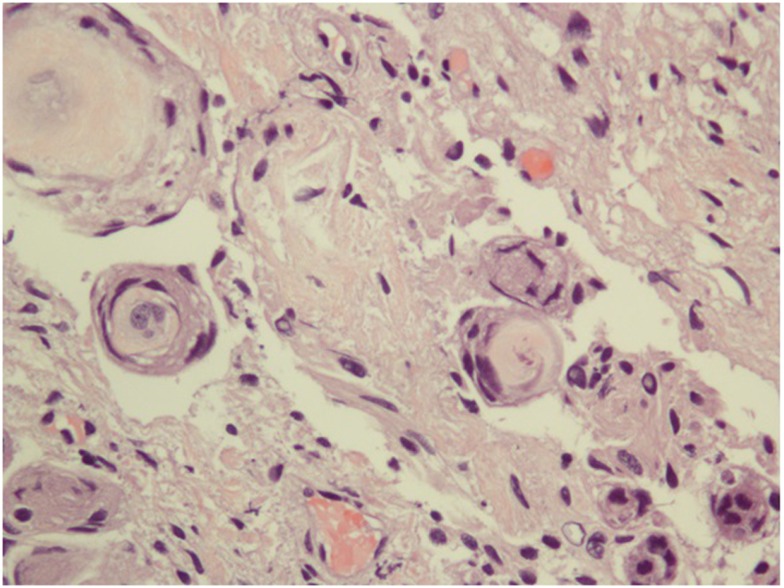
**The specimen consists of uniform, benign-appearing, spindle-shaped cells with whorl formations containing hyalinized material with some degeneration**. In time, with calcification, these whorls will progress to psammoma bodies classically observed in meningiomas.

**Figure 3:**
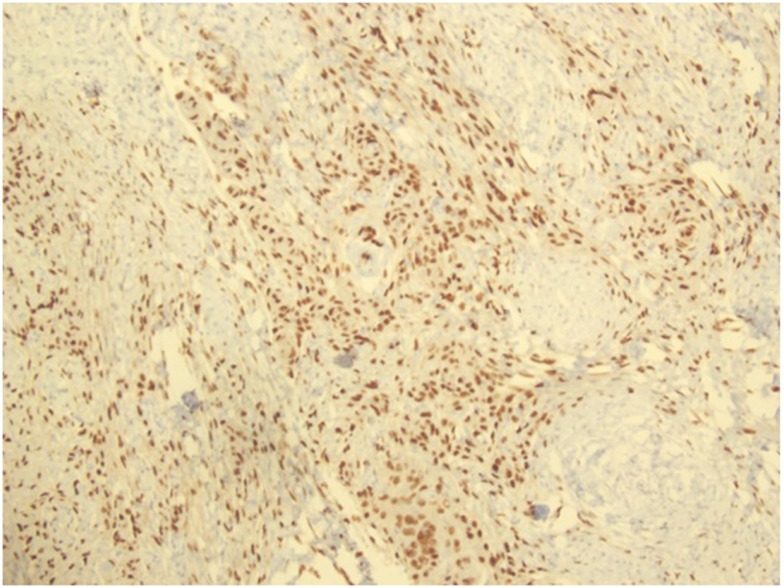
**Progesterone receptor antibody staining, showing the presence of PR in the specimen**.

At our institution, she received dexamethasone 4 mg every 6 h and had an uneventful delivery through a cesarean section (C-Section). Three days after C-section, a bicoronal craniotomy was done with subtotal tumor removal. A temporary lumbar drain was placed. Levetiracetam for seizure prophylaxis was given and dexamethasone taper was started on postoperative day 5. Surgical pathology showed meningioma WHO grade I, positive for progesterone receptors. The Ki-67 proliferation index overall was less than 1% (Figures 2 and 3). Following surgery, she was able to perceive light and motion with her OS.

## Discussion

The clinical presentation of unilateral optic atrophy with contralateral papilledema and anosmia was first fully described by Kennedy in 1911 (1). Mass lesions accounting for Foster Kennedy syndrome are frequently located in the olfactory groove, falx cerebri, sphenoid wing, or subfrontal region (2). Most masses are neoplastic, and meningiomas are the most prevalent lesions. Other reported mass lesions causing Foster Kennedy syndrome include frontal lobe abscesses, craniopharyngiomas, pituitary adenomas, plasmacytomas, nasopharyngeal angiofibromas, neuroblastomas, and aneurysms (2). Direct compression of one optic nerve accounts for ipsilateral optic atrophy. Raised intracranial pressure (ICP) causes contralateral papilledema that usually precedes the ipsilateral optic atrophy (3) However, bilateral optic nerve compression without elevation of ICP, and chronic elevated ICP with compression have been postulated as other possible mechanisms (4). Headaches, emotional lability, memory loss, nausea, vomiting, or weakness may also be present (4). Visual changes may present as transient visual obscurations, central scotomata, unilateral or bilateral visual loss, or visual field defects (4).

Meningiomas account for 15% of all intracranial tumors, and may present as a solitary mass or as multiple masses, the latter usually associated with neurofibromatosis type 2. Most common locations of meningiomas are over the convexities of the parasagittal sphenoid ridge, olfactory groove, suprasellar/parasellar regions, tentorium cerebelli, pineal region, optic nerve, and foramen magnum (5).

Olfactory groove meningiomas (OGM) account for 8–13% of all intracranial meningiomas (5). These tumors arise in the midline of the cribriform plate and the fronto sphenoidal suture, and may extend from the crista galli to the tuberculum sellae (6). Extension may be asymmetric and may compromise the ethmoid sinuses, the nasal cavity, or even the orbit. (7). OGMs receive their vascular supply primarily from the anterior and posterior ethmoidal arteries, branches of the ophthalmic artery.

Risk factors for development of meningiomas include chromosome deletion, previous ionizing therapy, and head trauma (5). The reported 2:1 female/male increases during reproductive years to 3.15:1 (8). Meningiomas may progress following contraceptive implants, during the luteal phase of the menstrual cycle, or during pregnancy (9, 10). Further studies have shown the presence of estrogen, progesterone, and androgen receptors, with progesterone receptors being present in 80% of meningiomas among women, and only 40% among men (11). However, the potential correlation of presence of hormone receptors and the natural history of meningiomas has been inconsistent.

Incidence of meningiomas in pregnancy is not different than in non-pregnant women. However, symptoms may flare up during the first two trimesters of pregnancy, with subsequent tumoral shrinkage post-partum and possible recurrence with subsequent pregnancies (12, 13). Besides the proposed influence of sexual hormones, this clinical flare-up has been attributed to water retention and engorged blood vessels (14, 15).

In pregnant patients suspected of having an intracranial mas lesion, MRI remains the preferred imaging modality because of its greater resolution, increased sensitivity, and lack of ionizing radiation. The use of gadolinium in pregnant patients controversial as gadolinium can cross the placenta, but has not been shown to cause birth defects (16, 17). CT carries the risk of ionizing radiation to the fetus, but given its greater availability, lower cost, and utility in assessing of calcifications, its use should be considered in selected circumstances and always with proper abdominal shielding (18, 19).

Management of meningiomas during pregnancy requires a multidisciplinary approach with close surveillance of mother and fetus, with delivery at term and post-partum tumor resection (20). Vaginal delivery is contraindicated in the presence of raised ICP, because cerebrospinal fluid (CSF) pressure increases with each uterine contractions during the second stage of labor (21). However, tumor resection might be warranted before delivery in patients with malignancy, active hydrocephalus requiring shunting, benign growth associated with signs of impending herniation, or progressive neurologic deficits (14). Peripartum and perioperatively edema leading to ICP can be managed with tapering doses of dexamethasone. (22, 23). Mannitol should be used with caution, given that it crosses the placenta and may affect the fetus. Its use should only be limited to emergency situations (24). If seizures occur, they should be treated appropriately, as the risk associated with recurrent seizures is far greater than the risks of properly chosen antiepileptic drugs (14). If tumor margins are not well defined, or the nature of the meningioma is more aggressive with the potential for high risk of recurrence, adjuvant external beam radiation in the early post operative period should be considered (21).

Olfactory groove meningiomas may grow rapidly during the first two trimesters of pregnancy leading to devastating consequences. Close monitoring with thorough neurologic examination is recommended as the first step to assess any significant changes in mass effect (25). The combination of unenhanced and enhanced MRI is recommended for the detection of residual or recurrent meningiomas after surgery.

Our patient presented with a symptomatic OGM that grew during her pregnancy. Following a successful delivery, the meningioma was removed.

## Conflict of Interest Statement

The authors declare that the research was conducted in the absence of any commercial or financial relationships that could be construed as a potential conflict of interest.
